# Epidemiology, Classification and Management of Undescended Testes: Does Medication Have Value in its Treatment?

**DOI:** 10.4274/Jcrpe.883

**Published:** 2013-05-30

**Authors:** Ayhan Abacı, Gönül Çatlı, Ahmet Anık, Ece Böber

**Affiliations:** 1 Dokuz Eylül University Faculty of Medicine, Department of Pediatric Endocrinology, İzmir, Turkey

**Keywords:** Undescended testes, treatment, human chorionic gonadotropin

## Abstract

Genetic, hormonal, and anatomical factors are believed to be involved in the etiology of undescended testes. Due to increased risk of infertility, testicular cancer, torsion and/or accompanying inguinal hernia (>90%) as well as cosmetic concerns, all these patients require treatment. In this review paper, we aimed to evaluate the success rates of treatment modalities used in undescended testes, beginning from 1930 to the present, and to draw attention to the possible risks and benefits and also the efficacy of hormonal therapy in the management of the disorder, which is still a controversial issue. Hormonal therapy may lead to penile growth, painful erection, and behavioral changes while on treatment. In recent years, it has been reported that human chorionic gonadotropin (hCG) treatment was associated with interstitial edema due to increased vascular permeability, inflammation-like changes, and several adverse effects on germ cells by increasing pressure and apoptotic process. It has also been reported that LHRH analogues have positive effects on germ cells by increasing fertility in patients undergoing unilateral or bilateral orchiopexy. In some studies, the success rate of hCG treatment was reported to be higher following buserelin. In some other studies, hCG treatment was recommended before orchiopexy to reduce the risk for surgical ischemia. There are a limited number of randomized controlled studies, so evidence showing the efficacy of hormonal therapy is insufficient. According to the 2007 Consensus Report of Nordic countries, it is recommended that surgery is the first-line treatment modality in undescended testes and that it should be performed by pediatric surgeons and urologists at the age of 6-12 months.

**Conflict of interest:**None declared.

## INTRODUCTION

Undescended testis is present in about 1-4.5% of newborns with a higher incidence in preterms (30-45%) (1,2). In infants born with undescended testes, the testes may descend into the scrotum in 75% of full-term neonates and in 90% of premature newborn boys in infancy, and the incidence decreases to 0.8-1.2% at 1 year of age (3,4,5). Undescended testis with ambiguous genitalia always needs immediate systematic work-up (6).

Undescended testes should be differentiated from retractile, ectopic, and vanishing testes. The differential characteristics of undescended and ectopic testes are summarized in Table 1 (4). Patients with undescended testes should be treated because of increased risk of infertility, testicular cancer, torsion and/or accompanying inguinal hernia (>90%), as well as because of cosmetic concerns (1,5,7).

In the scope of this review paper, we aimed to evaluate the success rate of treatment modalities applied in undescended testes, beginning from 1930 to the present, with possible risks and benefits and also to assess the efficacy of hormonal therapy in the management of the disorder, which is still a controversial issue. 

**Embryological Development of the Testes and Normal Testicular Descent Mechanism**

In the fourth to sixth week of pregnancy, primordial germ cells originating from embryonic yolk sacform the gonadal structure, moving forward to the gonadal ridge in the coelomic epithelium through amoeboid movements. Based on the presence of SRY gene in chromosome Y, primordial germ cells are differentiated into bipotential gonadal testicular or ovarian cells between the fourth to sixth weeks of pregnancy. By the 8th week of gestation, Sertoli cells secrete anti-Mullerian hormone (AMH), causing regression of Mullerian structures. By the 10^th^ week, Leydig cells of the fetal testis start secreting testosterone, stimulating the Wolffian duct to form the epididymis, vas deferens and seminal vesicles. During the 10-12^th^ weeks of pregnancy, testosterone is converted into dihydrotestosterone by 5-alpha reductase, resulting in virilization of the external genital region (4).

Testicular descent occurs in two distinct phases, namely, transabdominal and transinguinal. The transabdominal phase occurs between the 7^th^ and 15^th^ weeks of pregnancy (8). Transabdominal descent depends on insulin-like peptide (INSL3) and is related to the receptor leucine-rich repeat family of G-protein-coupled receptor 8 (LGR8), while inguinoscrotal descent is mediated by androgens (8,9). Mutations or polymorphism of INSL3 and LGR8 are uncommon causes of undescended testes (10). However, INSL3 may be important also in the second phase of testicular descent (9). Transinguinal phase, which follows transabdominal phase, is completed at the 35^th^ week of pregnancy (8). During this phase, the peritoneum grows into the gubernaculum to form the processus vaginalis (PV), which allows the intra-abdominal fetal testis to reach the subcutaneous site in the scrotum within a diverticulum of the peritoneum. This descent is believed to be indirectly controlled by the action of androgens on the genitofemoral nerve and the subsequent release of guiding neurotransmitters (4,11). Congenital undescended testes is often associated with hypogonadotropic hypogonadism, decreased Leydig cell function, and inadequate androgenic effect due to diseases such as androgen receptor defect. Smoking and environmental factors (such as exposure to endocrine disruptors) during pregnancy may also lead to undescended testes (8,12,13).

Examples of factors that have been proposed to influence testicular descent are given in Table 2 (9).

**Etiology and Classification**

Several factors including humoral and genetic components are involved in testicular descent (8). The risk of undescended testes is 10.1 fold higher in male twins if present in one of them, 3.5 fold higher in males with a brother with undescended testes, and 2.3 fold higher in males with a father with the condition (2). Undescended testis is a common finding among male disorders of sexual differentiation (DSD). Normal testicular descent is dependent on an intact hypothalamo–pituitary–testicular axis. Although the exact etiology is still unknown, it is postulated that genetic, hormonal (hypothalamic-pituitary-gonadal axis dysfunction, congenital hypogonadotropic hypogonadism, testicular dysgenesis), and anatomical (short vas deferens and spermatic vessels) factors are involved (2,6,14,15). A birth weight <2.5 kg, being small for gestational age, prematurity, low maternal estrogen levels, and placental insufficiency with decreased human chorionic gonadotropins (hCG) secretion are suggested as risk factors for undescended testes (6). In addition, exposure to environmental factors such as persistent exposure to organochlorine compounds, mono-esters of the phthalates, maternal smoking, and maternal diabetes mellitus are also reported to be risk factors for maldevelopment of the male reproductive organs (16). However, none of these factors has been shown to be solely responsible for the etiopathogenesis of undescended testes (8).

Several hormonal factors play important roles in testicular descent to the scrotum following the transinguinal phase (Table 2). Gonadotropins, hCG, AMH, androgens, and defensins have a critical effect in testicular descent. Dihydrotestosterone, rather than testosterone, is a key driver in testicular descent to the scrotum. This may explain the reason for undescended testes observed in the presence of 5-alpha reductase defect. Testicular descent mechanism is also affected adversely in states of hypothalamic-pituitary axis dysfunction. The higher prevalence of undescended testes in cases with Prader-Willi syndrome, Kallman syndrome, pituitary hypoplasia and anencephaly indicates the critical role of the hypothalamic-pituitary axis and it is suggested that the major cause of undescended testes is hypothalamic-pituitary axis dysfunction. This hypothesis is the main theme of hormonal therapy in the management of undescended testes. In addition, anatomical defects may also affect testicular descent adversely (short vas deferens and spermatic vessels) (4).

Undescended testis may be unilateral or bilateral, mostly involving the right side (70%). Classification is based on testicular location, which may be either along the normal line of descent (abdomen, inguinal canal, external ring, prescrotal, upper scrotal) or in an ectopic position (usually in the superficial inguinal pouch or perineal, rarely perirenal) (1). Clinical examination findings reveal that 80% of UDT are palpable and sit in the inguinal superficial pouch (30%), the inguinal canal (20%), the upper scrotum (45%) and rarely (5%) in the perineum or the thigh and that 20% of UDT are non-palpable and are located in the abdominal cavity (17).

**Diagnosis and Physical Examination**

The diagnosis of undescended testes is clinical. The examination should be performed by an experienced person and should always be performed using a two-handed technique (16,18). Palpation should take place in an anxiety-free medium and with warm hands, since cold or anxiety can cause the cremasteric reflex to retract the testes (16).

The patient should be examined in the supine position with legs abducted initially. The examination should begin with exploration of the undescended testes at the anterior superior iliac spine and sweep the groin from lateral to medial with the non-dominant hand. Once the testis is palpated, the examiner should grasp it with the dominant hand and continue to sweep the testis toward the scrotum with the other hand. Testicular mobility, size, consistency, and spermatic cord tension should be assessed. The position of the testis in the scrotum should be maintained for a minute, so that the cremaster muscle is fatigued. Then the testis is released, and if it remains in place for a short time but then retracts, it is considered retractile. In all patients, the size, location, and texture of the contralateral descended testes should also be checked. The key to distinguishing a retractile from an undescended testis is success of delivery and stability of the testis within the scrotum. The retractile testis will remain intrascrotal after overstretching of the cremaster muscle, whereas a low undescended testis will return to its undescended position after being released (1).

**Imaging Techniques and Laboratory Tests**

Nearly 20% of undescended testes are impalpable. There are several reasons for impalpable testes, including intraabdominal, intracanalicular or ectopic location of the testes, testicular dysgenesis and absence of the testes. The use of imaging techniques in the diagnosis of impalpable testes is controversial. Today, it is recommended that impalpable testes should be examined by laparoscopic surgery with or without radiological guidance (11).

Imaging techniques include ultrasonography (USG), computerized tomography (CT), and magnetic resonance imaging (MRI). MRI angiography has been reported to have low diagnostic accuracy (1,11). CT is the least preferred technique. Although several imaging options are available, none of them gives accurate results for testicular morphology and position. The diagnostic accuracy rate of imaging techniques has been reported to be 44% (11).

USG is a useful technique in cases which are scheduled for laparoscopic or inguinal intervention due to impalpable testes. However, it has a limited diagnostic role, since 70% of palpable testes which do not move down into the scrotum can be diagnosed using USG, but only 12% of impalpable testes which have been operated on had been diagnosed by using USG (1). In a study, the sensitivity of USG for the diagnosis of inguinal undescended testis was reported to be 95-97% (1). In another study, it was emphasized that ultrasound does not reliably localize non-palpable testes and does not rule out an intraabdominal testis (19). Others have also stated that the best evaluation method for impalpable testes is physical examination and that USG has no superiority (1,11).

MRI is a useful technique particularly for ectopic testes located in the abdomen, which cannot be detected by using laparoscopic or open surgery (1). Due to its superiority in differentiation, MRI is the most commonly used method to differentiate testicular tissue from the adjacent tissues in obese patients. However, unlike USG, it is not sensitive in detection of abdominal testes (11). In a study including 56 patients who were radiologically assessed before surgery, it was reported that the sensitivity and specificity of USG was 76% and 100%, respectively (diagnostic accuracy: 84%), whereas the sensitivity and specificity of MRI was 86% and 79%, respectively (20).

Gadolinium-enhanced MRI (Gd MRI) angiography is a reliable method in the differential diagnosis of vanishing testes from impalpable intraabdominal and intracanalicular testes. It has been emphasized that Gd MRI angiography may reduce unnecessary laparoscopic procedures and may provide insight on which surgical approach is performed. A total of 100% of intracanalicular testes and 96% of intraabdominal testes can be detected using Gd MRI angiography (11). However, it has also been reported that this technique is less commonly preferred due to requirement for sedation and high cost (21).

In conclusion, in patients with undescended testes who cannot be clinically diagnosed, the primary imaging technique should be USG, while MRI should be the secondary modality. Inguinal exploration or laparoscopic intervention should be considered in patients who cannot be radiologically diagnosed (11).

Endocrinological and chromosomal investigations should be performed in cases of bilateral impalpable undescended testes accompanied by hypospadias. Anorchia should be considered in cases with bilateral impalpable testes when postnatal physiological testosterone peak or testosterone response to hCG test at >3 months postnatally are lacking. AMH and inhibin B levels may be tested to investigate whether testes are present (1). The positive predictive value of the hCG test was found to be 89%, while its negative predictive value was 100% in patients with bilateral undescended testes (22).

**Testicular Biopsy**

Testicular biopsy during orchiopexy is recommended for patients with undescended testes accompanied by abnormal genital structure and chromosomal disorder (8).

**Histology**

It has been reported that in patients with undescended testes, testes are histologically normal at birth and that the histological changes occur after 6-12 months. These changes include delayed germ cell maturation, decreased germ cell number, and hyalinization of seminiferous tubules (4,23).

**Undescended Testes and the Rationale for Treatment**

**1- Risk for Infertility**

Ten percent of infertile males have a history of undescended testes. The infertility risk is sixfold higher in patients with bilateral undescended testes compared to patients with unilateral undescended testis or with a healthy population (7). In unilateral undescended testis, although one testis descends in early term, the number of germ cells is lower in these patients compared to the healthy population due to intrinsic pathology (testicular dysgenesis). Several histological changes in the contralateral testis, which is in its normal scrotal location, have been observed in patients with unilateral undescended testis (shared intrinsic pathology) (23). Delay in Ad (dark) spermatogonia transformation is seen in scrotal testis as well since Leydig cell dysfunction is present in cases with hypothalamo-pituitary hypogonadism or testicular dysgenesis. This is known as shared intrinsic pathology. Therefore, the risk of infertility is increased in patients with unilateral or bilateral undescended testes and is higher in patients left untreated (24).

It has been reported that the rate of azoospermia is 13.3% and 88.6% in patients with unilateral and bilateral untreated undescended testes, respectively. In bilateral undescended testes, the risk of azoospermia decreases to 32% among patients treated medically and to 46% in patients who underwent orchiopexy as a child. In unilateral cryptorchid patients, the incidence of azoospermia (13%) was found to be similar in treated and untreated patients, regardless of treatment (25). Tasian et al (26) emphasized the importance of early treatment after detecting that the rates of germ cell loss and Leydig cell loss were 2% and 1%, respectively, per month in untreated cases. However, infertility was reported in 1/3 (44/135) of cases with undescended testes despite orchiopexy (54% in bilateral undescended testes and 9% in unilateral undescended testis). Follicle-stimulating hormone (FSH) levels were within normal range in 45% of these cases (27). Hadziselimovic et al (25) also showed that infertility was present in 35% of the patients, despite normal number of germ cells and early orchiopexy (<6 months).

**2- Risk for Cancer**

The risk for cancer is 35 to 48 times higher in patients with undescended testes compared to the overall population (5).The risk for malignant degeneration is 3-18% in these patients (28). A total of 10% of testis malignancies are associated with undescended testes (5,18,23). The risk for malignant degeneration is sixfold higher in patients with abdominal testes (5,23). Although some authors reported that the risk for malignancy cannot be reduced by early orchiopexy (5,17), it has also been reported that the risk for malignancy is increased sixfold in patients who do not undergo orchiopexy in the prepubertal period or in patients with delayed surgery (29). Malignancy risk is 32 times higher in patients undergoing orchiopexy later than age 11 years (5,23,30). The age range during which testis tumors most frequently develop in these cases is 20-40 years (2). The most common types of testicular cancer encountered are seminoma and embryonal carcinoma (23).

**3- Risk for Torsion**

The risk for torsion is higher in adult patients with undescended testes compared to overall population. A germ-cell tumor was reported to occur in 64% of such cases. It was also suggested that the risk for torsion was associated with the duration of the undescended testes (29).

**Undescended Testes and the Most Appropriate Age for Treatment**

Undescended testes often resolve spontaneously as a consequence of the effect of luteinizing hormone (LH) and FSH peak which occurs during the mini-puberty period (1,5). As a result, it is recommended that medical or surgical treatment should be initiated after the age of 6 months. Several treatment protocols have been proposed for treatment of undescended testes. The success rates of these modalities depend on the treatment options (dose and duration), age of the patient, position of testes, and unilateral or bilateral nature of the disease.

There are two treatment approaches for undescended testes: hormonal and surgical (31). The treatment of choice is hormonal therapy in Europe since 1930s, while surgery is preferred in the first-line setting in the USA (1,31). The use of hormonal therapy is still controversial in the literature; however, it has been widely used in many centers. Although the time for surgery is also still a matter of debate, it is generally recommended that surgery should be performed at 3, 6, 9 or 12 months (2,7). Cortes et al (32) reported that there were several adverse effects of hormonal therapy on germ cells in patients who received medical treatment over the age of one year, and advocated that hormonal therapy should not be administered between 1-3 years of age. According to the 2007 Consensus Report of Nordic countries including Sweden, Ireland, Denmark, Norway, and Finland on management of undescended testes, it is recommended that undescended testes should be surgically descended to the scrotum at 6-12 months (8). According to the results of a recent questionnaire about management of undescended testes given to pediatric endocrinologists in Turkey, 58.3% of the participants favored medical treatment, while the remaining 41.7% preferred surgical treatment for management of undescended testes. Of those who advocated surgery, 56% (n=14) believed that the appropriate time for orchiopexy was between 6-12 months of age, while another 32% (n=8) suggested the age of 12-24 months. The localization and uni/bilaterality of the undescended testes were important points that affected medical therapy decision. For intraabdominal testes, 80.9% of the participants suggested surgical therapy and the remainder suggested medical therapy initially. For bilateral inguinal testicles, 63.6% of the questionnaire participants suggested medical therapy, however, the other 36.3% preferred orchiopexy without considering whether the condition was bilateral or unilateral.

**Undescended Testes and Medical Treatment**

Although the use of hormonal therapy is still controversial in the literature, it has been used in Europe since 1930s (31).

Drugs and drug combinations used for medical treatment include androgens (testosterone), hCG, gonadotropin-releasing hormone (GnRH), hCG+GnRH, hCG+FSH, and human menopausal gonadotropin (hMG).

The rationale for hormonal therapy is based on a consideration of the etiological factors in the development of undescended testes (31). Although the mechanism of effect of gonadotropins on postnatal testicular descent is still unclear, it has been suggested that the spermatic cord and/or the cremasteric muscle may be involved. Results of trials with monotherapy or combination therapies have been reported. The success rate of these therapies depends on the position of the testes, inclusion criteria, and type and dosage of these drugs. The most common cause for treatment failure is anatomical position (inguinal hernia, abnormal testis-epididymis fusion, etc) (33). The opinions of pediatric endocrinologists in Turkey with reference to hormonal therapy was mentioned above.

**hCG Treatment and Recommended Doses**

The most frequently used hormonal therapy for undescended testes is hCG both in Turkey and worldwide; 90% of Turkish pediatric endocrinologists who suggested medical therapy preferred hCG. The success rate of this therapy ranges from 0 to 55%, while the success rate of GnRH ranges between 9 and 78% (34). The success rate may be higher in several studies, due to inclusion of cases with retractile testes in the study group (5). Age of treatment is also a major factor influencing the success rate (34). de Muinck Keizer-Schrama et al (35) reported that the highest success rate was obtained at ages 5-12 years. However, other studies reported that success rates were maximal at ages 2-5 years (36). It is also suggested that treatment should be initiated before the age of 2 years, since several histological changes may already be seen at this age (34). In a meta-analysis (37) assessing the efficacy of hormonal therapy (1985-1990), the success rates of LHRH and hCG were detected to be 47% and 33% in 22 nonrandomized studies, 21% and 19% in 11 randomized studies, respectively, whereas the success rate of placebo was 4%. There was no significant difference in the success rates of treatments between patients younger or older than 4 years.

Aycan et al (14) analyzed the responses to hCG treatment in two groups of patients with undescended testes (mean age 5.2±3.1 years) who were randomized to high-dose hCG (1500 IU/m2/week for three weeks) or low-dose hCG (500 IU/m2/week for three weeks). There was no statistically significant difference in treatment responses between the two groups; however, the success rate of low-dose hCG was slightly higher compared to the high-dose therapy (66.7% vs. 57.1%) (14). In Turkey, modalities and duration of medical therapy were found to vary according to institutions. Fifty percent of the questionnaire participants suggested a hCG dose recommended by the World Health Organization (WHO) (23), while 31.8% suggested hCG at a dose of 1500 IU/m2/week.

Recommended hCG doses for treatment of undescended testes are as follows (23,38):

1- According to the WHO:

250 IU in boys <1 year of age twice a week for five weeks

500 IU in those of ages 1-5 years twice a week for five weeks

1000 IU in those of ages >5 years twice a week for five weeks

2- The other recommendations are:

1500 IU/m2/week twice a week for 4-9 weeks (with a total maximum dose of 10 000 IU)

Four injections of 100 IU/kg at 4- to 5-day intervals

Seven injections of 1500 IU, every other day

It has been also been reported that the testes return to their initial suprascrotal position in 25% of the patients who are responsive to hCG treatment (39).

Possible side effects of hCG treatment include penile growth, appearance of pubic hair, painful erection, behavioral changes, transient inflammatory changes in the testes, germ cell apoptosis, and decreased testicular volume in adulthood (39,40).

**Combination Therapy**

There are reports of several studies investigating the success rate and efficacy of hCG in combination with hMG or GnRH analogues (31). In these studies, hMG 75 IU/week for 6 weeks and GnRH 1200 µg/day (1.2 mg/day) for 28 days nasally were used in combination with hCG treatment (31).

In a study investigating the efficacy of combination therapy in the management of undescended testes, Bertelloni et al (31) reported that the success rate was below 20% for both short- and long-term treatment (18.9% for hCG; 12.8% for GnRH; 15% for hCG in combination with GnRH; 12.8% for hCG in combination with hMG given long-term). Although some authors suggest that there is no significant difference in the success rates of these treatments applied in unilateral and bilateral undescended testes, others suggest opposing arguments (5). In a multi-center study comparing the efficacy of hCG and intranasal GnRH, it was detected that hCG treatment was more effective in patients with bilateral undescended testes compared to those with unilateral testis (23% vs. 19%). The authors also recommended hCG treatment as the first-line treatment in prepubertal cases below the age of 1 year due to its higher efficacy rate (36). In another randomized, double-blind study investigating the efficacy of hCG (300 IU per week for 4 weeks) and GnRH analogues (1.2 mg/day for 28 days), it was detected that GnRH analogues were more effective than hCG treatment in 33 patients of ages 1-5 years with undescended testes (29 unilateral, 4 bilateral). However, there was no statistically significant difference (19% vs. 6%; p=0.23). It was also suggested that the higher success rate found in other studies might be explained by inclusion of patients with retractile testes in the series (41).

In another combination therapy study, hCG was given as 500-2000 IU, twice a week for 6 weeks and FSH as 75 IU/at least once a week for 6 weeks. The success rate of this therapy may vary depending on the patient’s age. The success rate was found to be 13.3% for boys with unilateral undescended testis of ages between 1 and 2 years, 29.6% for those of ages 3-4 years, 38.2% for those of ages 5-6 years, and 50% for those of ages 7-11 years. The success rate was 16.6% for boys with bilateral undescended testes aged 1-2 years, 27.2% for those of age of 3-4 years, and 37.5% for those of ages 7-11 years (42).

**Hormonal Therapy and Side Effects**

Hormonal therapy is often a safe treatment modality; however, it may lead to penile growth, painful erection, and behavioral changes while on treatment (31). In recent years, it has been reported from experimental and human studies that hCG treatment caused interstitial edema due to increased vascular permeability, inflammation-like changes (leukocyte extravasations), and several adverse effects on germ cells by increasing pressure and apoptotic process (39,43,44,45,46). It has also been shown that the incidence of inflammatory changes following hCG treatment is higher in patients with abdominal testes compared to scrotal testes (46).

Although there are several publications showing that the effects of hormonal therapy on germ cells were transient (acute effect) (47,48), in a human study by Dunkel et al (39) it was demonstrated that the rate of apoptosis was 3-4 times higher in the hCG treatment group 20 years later compared to those who did not receive treatment. The authors reported that testicular volume was reduced and FSH levels were higher in patients who received hCG treatment during their prepubertal period, indicating that the treatment can exert a long-term (chronic) effect (39).

In recent years, a number of studies have been published indicating the adverse effects of medical treatment on germ cells. However, it has also been reported that preoperative and postoperative administration of LHRH analogues have positive effects on germ cells by increasing fertility in patients undergoing unilateral or bilateral orchiopexy (5,45,49). In another study, low-dose LHRH was observed to have a positive effect on the spermatogonia/tubule ratio (45,50). In a randomized controlled study investigating the effect of nasal buserelin (20 µg/day), it was observed that the rate of germ cell maturation index was higher in patients with undescended testes compared to the placebo group. The authors also observed that the success rate of hCG treatment following buserelin was higher, despite no additional effect on germ cell maturation (50). Several studies also recommended hCG treatment before orchiopexy to reduce the risk for surgical ischemia (51).

Some authors have also pointed out the paucity of randomized controlled studies with adequate sample sizes and adequate statistical power in the literature; thus, there is insufficient evidence showing the efficacy of hormonal therapy on undescended testes (34). According to the 2007 Consensus Report of Nordic countries, it is recommended that surgery is the first-line treatment modality, and pediatric surgeons and urologists should perform surgery at age 6-12 months (8).

**Undescended Testes and Surgery**

The success of surgery is defined as presence of testes in the scrotum without testicular atrophy and/or any recurrence for ≥1 year. It has also been reported that surgery is not totally safe; the complication rate ranges from 1.5% to 12%. Surgery may be complicated and may result in higher complication rates in cases when the scrotum is undeveloped and the testis is malformed, small, and associated with short vessels (34). Ritzen et al (52) recommended hormonal therapy as the first-line treatment and immediate orchiopexy as the second-line treatment if medical treatment fails. According to these authors, hormonal therapy before orchiopexy increases the blood flow in this region and facilitates the surgical intervention by relaxing the cremaster muscle (52).

In 64 articles analyzing 8425 patients with undescended testes, it was reported that the success rate of surgery performed by a skillful surgeon was 74% for abdominal testes, 87% for intracanalicular testes, and 92% for undescended testes in the external inguinal canal (53). In another study, the success rate of orchiopexy was reported to be >95% in inguinal testes and between 85% and 90% in abdominal testes (54).

Palpable testes following surgery should not be considered as functional testes. Hormonal production and spermatogenesis should be normal to consider the testes as functional. The age of the patient during orchiopexy is also of great importance for sperm quality (55). Despite surgical treatment by orchiopexy, the long-term outcome still remains problematic and controversial. Impaired fertility (33% in unilateral cases and 66% in bilateral undescended testes) and a cancer risk 5-10 times greater than normal is observed over time (2). Hadziselimovic et al (56) reported infertility in 35% of the patients with undescended testes with normal germ cell number before surgery despite early orchiopexy performed under age 6 months. They suggested that this might be explained by defective transformation of germ cells due to lack of a mini-pubertal period (56). During mini-puberty, progenitor spermatozoa are transformed into Ad (dark) spermatogonial cells owing to the peak effect of LH and testosterone, an effect which occurs particularly in the postnatal 2-3 months (24,56). The infertility rate may increase up to 90% in patients who have not undergone a mini-pubertal period (56).

Testicular retraction or atrophy has been reported to occur in a frequency of 0-2%, and postoperative hernia has been noted in 2-3% of cases after orchiopexy (1). In addition, surgery may result in Sertoli and Leydig cell dysfunctions. Major complications include pain, hematoma, infection, and anesthetic side effects. Testicular atrophy and vas deferens damage are the most frequently seen intraoperative complications (8).

In conclusion, the success rate of surgery is 90%, whereas the reported success rate of hCG treatment by randomized studies is 19-25% (27,30). It is difficult to establish efficacy and safety of hormonal therapy due to the limited number of randomized controlled studies with long-term follow-up and due to the controversial data in the literature. Most of undescended testes descend into the scrotum during the first 3 months. Thus, at present, there is a consensus that the diagnosis should not be definitely established before 6 months of age. Based on the recent literature data and consensus reports, it is recommended in the 2007 Consensus Report of Nordic countries that surgery should be the first-line treatment modality and should be performed at age 6-12 months. 

## Figures and Tables

**Table 1:**
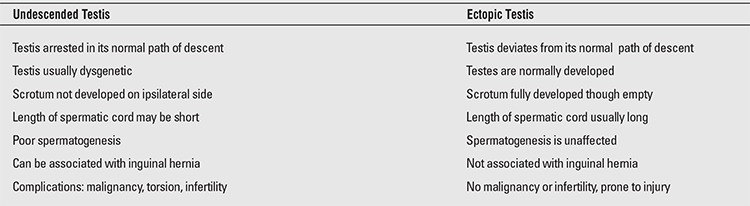
Comparison of undescended with ectopic testis (4)

**Table 2:**
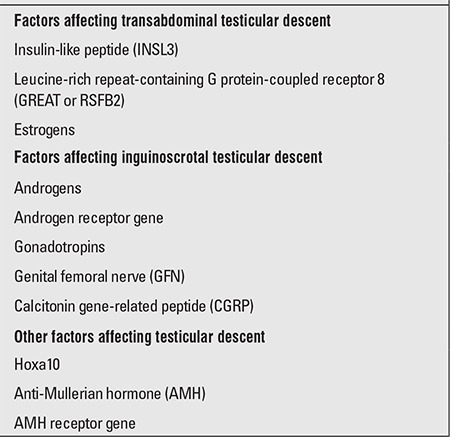
Examples of factors that have been proposed to influencetesticular descent (9)
